# lncRNA-SNHG14 Plays a Role in Acute Lung Injury Induced by Lipopolysaccharide through Regulating Autophagy via miR-223-3p/Foxo3a

**DOI:** 10.1155/2021/7890288

**Published:** 2021-09-08

**Authors:** Jun Hong, Shijing Mo, Fangxiao Gong, Zongbin Lin, Hanhui Cai, Ziqiang Shao, Xianghong Yang, Renhua Sun, Qiangnu Zhang, Jingquan Liu

**Affiliations:** ^1^Department of Intensive Care Unit, Zhejiang Provincial People's Hospital (People's Hospital of Hangzhou Medicine College), Hangzhou 310014, China; ^2^Department of Key Laboratory of Tumor Molecular Diagnosis and Individualized Medicine of Zhejiang Province, Hangzhou 310014, China; ^3^Department of Hepatobiliary and Pancreas Surgery, Shenzhen People's Hospital, Shenzhen, 518020 Guangdong, China

## Abstract

lncRNAs play important roles in lipopolysaccharide- (LPS-) induced acute lung injury. But the mechanism still needs further research. In the present study, we investigate the functional role of the lncRNA-SNHG14/miR-223-3p/Foxo3a pathway in LPS-induced ALI and tried to confirm its regulatory effect on autophagy. Transcriptomic profile changes were identified by RNA-seq in LPS-treated alveolar type II epithelial cells. The expression changes of lncRNA-SNHG14/miR-223-3p/Foxo3a were confirmed using qRT-PCR and west blot. The binding relationship of lncRNA-SNHG14/miR-223-3p/and miR-223-3p/Foxo3a was verified using dual-luciferase reporter, RNA immunoprecipitation, and RNA pull-down assays. Using gain-of-function or loss-of-function approaches, the effect of lncRNA-SNHG14/miR-223-3p/Foxo3a was investigated in LPS-induced acute lung injury mice model and in *vitro*. Increasing of lncRNA-SNHG14 and Foxo3a with reducing miR-223-3p was found in LPS-treated A549 cells and lung tissue collected from the LPS-induced ALI model. lncRNA-SNHG14 inhibited miR-223-3p but promoted Foxo3a expression as a ceRNA. Artificially changes of lncRNA-SNHG14/miR-223-3p/Foxo3a pathway promoted or protected cell injury from LPS *in vivo* and in *vitro*. Autophagy activity could be influenced by lncRNA-SNHG14/miR-223-3p/Foxo3a pathway in cells with or without LPS treatment. In conclusion, aberrant expression changes of lncRNA-SNHG14 participated alveolar type II epithelial cell injury and acute lung injury induced by LPS through regulating autophagy. One underlying mechanism is that lncRNA-SNHG14 regulated autophagy by controlling miR-223-3p/Foxo3a as a ceRNA. It suggested that lncRNA-SNHG14 may serve as a potential therapeutic target for patients with sepsis-induced ALI.

## 1. Introduction

The prevention and treatment of sepsis complicated by multiple organ dysfunction are still facing enormous challenges. Acute lung injury (ALI) is a complication that occurs early and has a higher incidence in patients with sepsis [1]. Severe ALI can develop into acute respiratory distress syndrome (ARDS). The mortality rate of sepsis patients after developing ARDS ranges from 70% to 90% [2]. Reports have shown that even if the patients survive, the length of their hospitalization increases significantly, which also doubles the medical expenses [3]. Therefore, it is very crucial to prevent acute lung injury caused by sepsis.

Alveolar type II epithelial cells are important components that maintain the structure and function of the alveoli, and these cells participate in the repair of the alveoli after injury [4]. As such, the injury mechanism of alveolar epithelial cells in sepsis and the related protective measures have recently become a hot research topic. Through RNA sequencing, we found that LPS-treated alveolar type II epithelial cells had a significant decrease in miR-223-3p and a significant increase in their target gene, Foxo3a, which is an important gene for autophagy regulation [5]. In the present study, similar miR-223-3p and Foxo3a level changes were also found in mouse model of lipopolysaccharide- (LPS-) induced acute lung injury. Therefore, we speculated that the miR-223-3p/Foxo3a pathway may play a role in regulating autophagy during the process of LPS-induced alveolar type II epithelial cell injury. In recent years, autophagy and its role in sepsis complicated by ALI have gradually attracted the attention of researchers. But in the process of ALI caused by sepsis, the changes in autophagy in alveolar type II epithelial cells and the associated regulatory mechanisms are still unclear and need to be further explored. Previous studies have suggested that the role of autophagy in the occurrence of ALI is a double-edged sword in regard to alveolar epithelial cell injury. Inhibition of autophagy is not beneficial for cells to adapt to stress caused by inflammatory cytokines, and it reduces the chance that cells will be able to eliminate unfavorable factors [6, 7]. However, excessive activation of autophagy also leads to nonapoptotic programmed cell death [8, 9]. LPS-induced inflammatory response is a stress condition for cells that activate autophagy. Accumulating evidence has reported that in LPS-induced injury models, autophagy is an important initiation factor for cell death [10, 11]. However, other studies suggested that activating autophagy can protect cells treated with LPS [12]. Therefore, the intervention of autophagy level under the condition of LPS may contribute to reduce the cell damage caused by LPS. However, the concentration of LPS in the environment and the different pathophysiological processes of the disease are confounding factors that must be considered.

Long noncoding RNAs (lncRNA) are a type of RNA with lengths exceeding 200 nucleotides that are not translated into protein. It participates in the regulation of protein-coding genes via epigenetic regulation, transcriptional regulation, posttranscriptional regulation, etc. [13]. Emerging evidence has confirmed the relevance of long noncoding RNAs (lncRNAs) to ALI; thus, targeting lncRNAs has been highlighted as a novel therapeutic strategy for ARDS [14, 15]. Here, we found that lncRNA-SNHG14 was highly expressed during LPS-induced injury in alveolar type II epithelial cells. A previous study has shown that lncRNA-SNHG14 is involved in LPS-induced ALI [16]. However, more details about the role lncRNA-SNHG14 plays in ALI need further exploration. We showed that lncRNA-SNHG14 and miR-223-3p had complementary regions, suggesting that lncRNA-SNHG14 may act as competing endogenous RNA (ceRNA) to regulate miR-223-3p. Thus, this study explored the role and mechanism that the lncRNA-SNHG14/miR-223-3p/Foxo3a pathway plays in autophagy regulation during LPS-induced ALI.

## 2. Method and Materials

### 2.1. Cell Culture and ALI Cell Model Induced by LPS Exposure

Human ACE II-derived tumor A549 cell lines were acquired from American Type Culture Collection (ATCC, Manassas, VA, USA). A549 cells were cultured in DMEM supplemented with 10% fetal bovine serum (FCS) and 1% penicillin-streptomycin at 37°C. The cell culture condition was 5% CO_2_ and 95% air at 37°C. To simulate sepsis injury in vitro, cells were treated with 50 *μ*g/mL LPS for24 h. To activate or inhibit autophagy, cells were treated 100 nM rapamycin or 3 mM 3-methyladenine (3-MA) for 24 h, respectively.

### 2.2. ALI Mouse Model Induced by LPS Intratracheally Instilling

Specific pathogen-free mice (6-8 weeks old, 20-24 g) were randomly assigned into the control (*n* = 20) and ALI (*n* = 100) groups. In ALI groups, mice were intraperitoneally anesthetized with 1% pentobarbital. Then, LPS diluted in 100 *μ*L sterile normal saline was injected into the trachea through surgical treatment. The mice in the control group received the same surgical treatment but only received an injection of 100 *μ*L sterile normal saline without LPS. To artificially regulate the expression of lncRNA-SNHG14, miR-223-3p, or Foxo3a in the lung tissue of ALI mouse model, Adeno-Associated Viral (AAV) vectors containing shRNA-SNHG14 (*n* = 20), AAV-miR-223-3p overexpression vectors (*n* = 20), AAV-Foxo3a overexpression vectors (*n* = 20), and empty AAV vectors (*n* = 20) were administered by intranasal delivery 2 weeks before LPS instillation. All surgical and care procedures were conducted according to the National Institutes of Health Guide for the Care and Use of Laboratory Animals and approved by the Institutional Animal Care and Use Committee of the Zhejiang Provincial People's Hospital.

### 2.3. Lung Injury Estimation in ALI Mouse Model

After 12 h LPS, exposure bronchoalveolar lavage fluid (BALF) and lung tissue from ALI and control mice were collected and prepared for lung injury estimation. In brief, IL-6, IL-1*β*, and TNF-*α* levels in BALF were detected by enzyme-linked immunosorbent assay (ELISA). The number of cells in BALF was counted under a microscope. The percentage of neutrophils in BALF was calculated by Giemsa staining. Morphological changes in lung tissues are determined by hematoxylin-eosin (HE) staining. The wet (*W*) weight and dry (*D*) weight ratio were measured to indicate the pulmonary edema.

### 2.4. RNA Sequence

The total RNA was extracted from 24 h LPS-treated A549 cells. mRNA, lncRNA, and microRNA libraries were prepared. Paired-end sequences were performed in HiSeq system (Illumina, San Diego, CA).

### 2.5. Cell Transfection

pcDNA3.1-SNHG14 and pcDNA3.1-Foxo3a vectors were constructed and transfected into A549 cells to overexpress lncRNA-SNHG14 and Foxo3a, respectively. Empty pcDNA3.1 plasmids were used as control. To silence lncRNA-SNHG14, antisense oligonucleotide was transfected. To regulate the expression of miR-223-3p, miR-mimic, and miR-inhibitors were transfected with Allstar sequence and Miscript™ Inhibitor Negative Control as transfection control. Lipofectamine 3000 and Lipofectamine RNAimax were used for transfecting plasmid and oligonucleotide, respectively. The effect of the transfection was verified by qRT-PCR 48 h after transfection (Figure S1).

### 2.6. Cell Viability Assay

LPS treated or transfected cells were seeded in a 96-well plate containing 2 × 10^4^ cells/well and cultured for 24 h. Then, 100 *μ*L 1 : 10 CCK-8 reagent was added and incubated for 1 h. The absorbance value of each well was analyzed with a microplate reader at a wavelength of 450 nm.

### 2.7. Apoptosis Assay

Cells were plated in 96-well plates and treated with indicated transfection contents or/and LPS. After 24 h of treatment, cells were incubated with prepared 2× detection reagent provided by RealTime-Glo™ Annexin V Apoptosis Assay Kit. Luminescence was detected using a fluorescence plate reader to indicate apoptosis.

### 2.8. Quantitative Reverse Transcription Polymerase Chain Reaction (qRT-PCR)

Total RNA was extracted using Trizol reagent. For microRNA detection, total RNA was reversely transcribed into cDNA using miScript II RT Kit (QIAGEN, USA). cDNA was amplified using miScript SYBR Green PCR Kit. Primers for miR-223-3p and RNU6B were provided by miScript Primer Assays kit. For Mrna or lncRNA measurement, cDNA was synthesized using PrimeScript RT Reagent Kit. Then, qRT-PCR was performed using SYBR® Premix Ex TaqTM II reagent kit. Primers for Foxo3a and lncRNA-SNHG14 detection were as follows: Foxo3A, forward 5′-TGCGTGCCCTACTTCAAGGATAA-3′, reverse 5′-ACAGGTTGTGCCGGATGGA-3′; SNHG14, forward 5′-ACCTGCAAGCTTTTTGACCC-3′, reverse 5′-AGCAGACAAAGAAAAACCCCAAT-3′; and GAPDH, forward 5′-CCGCATCTTCTTGTGCAGTG-3′, reverse 5′-CCCAATACGGCCAAATCCGT-3′. Relative quantification was calculated using the 2^-△△CT^ method.

### 2.9. Western Blot

Total protein was isolated from tissues or cells by RIPA buffer and quantified using a BCA Protein Detection Kit. Then, 30 *μ*g protein was loaded to 12% SDS-PAGE gel for separation, following transfer to nitrocellulose membranes. 5% nonfat milk was used to block nonspecific positions. The membranes were incubated at 4°C overnight with primary antibodies against FoxO3a, LC3B, p62, and GAPDH primary antibodies. Following incubation with IgG HRP-linked secondary antibodies, membranes were treated with ECL Reagent (Beyotime) to visualize the immunoreactive bands.

### 2.10. Dual-Luciferase Reporter Assay

Wild-type (wt) SNHG14 sequence and mutant (mut) sequence as well as wt FOXO3a 3-untranslated region (3-UTR) sequences and mutant sequences were inserted into the pmirGLO luciferase reporter vectors. Reporter vectors were cotransfected with miR-223-3p mimic and Allstar sequence into A549 cells for 48 h. Dual-luciferase reporter assay kits were used to determine the luciferase activity.

### 2.11. RNA Binding Protein Immunoprecipitation (RIP)

Imprint® RNA Immunoprecipitation Kit was used to perform RIP assay according to the instruction provided by the manufacturer. In brief, A549 cells were lysed by RIP lysis buffer. Argonaute2 (Ago2) antibody was used for immunoprecipitation, and immunoglobulin G (IgG) was served as NC. The purified RNAs were then subjected to qRT-PCR analysis.

### 2.12. RNA Pull-Down Analysis

lncRNA-SNHG14 was transcribed and labeled with biotin *in vitro* using Biotin RNA Labeling (ROCHE) and T7 RNA polymerase (ROCHE) kit. After purification of the products, streptavidin agarose beads were added and further incubated at 4°C for 1 hour. The protein lysates of A549 cells were incubated with a mixture of streptavidin agarose beads and biotin-labeled SnHG14 at 4°C for 1 h to form the RNA-RNA/protein complex. AGO2 protein level was detected by Western blot, and the miR-223-3p level was detected by qRT-PCR analysis.

### 2.13. Enzyme-Linked Immunosorbent Assay (ELISA)

The concentrations of IL-6, IL-1*β*, and TNF-*α* in the culture supernate and BALF were measured using commercial ELISA kits (Invitrogen). All protocols were conducted according to manufacturer instructions.

### 2.14. Statistical Analysis

Statistical analyses were performed using the R software (Version3.5.1). Data were presented as mean ± standard deviation. Differences between groups were analyzed using a *t*-test for two groups and one-way ANOVA test followed with LSD test for more groups. A value of *P* < 0.05 was considered to be statistically significant.

## 3. Results

### 3.1. Changes in the lncRNA-SNHG14/miR-223-3p/Foxo3a Expression Level in LPS-Treated Alveolar Type II Epithelial Cells and Mouse ALI Models

**Figure 1:**
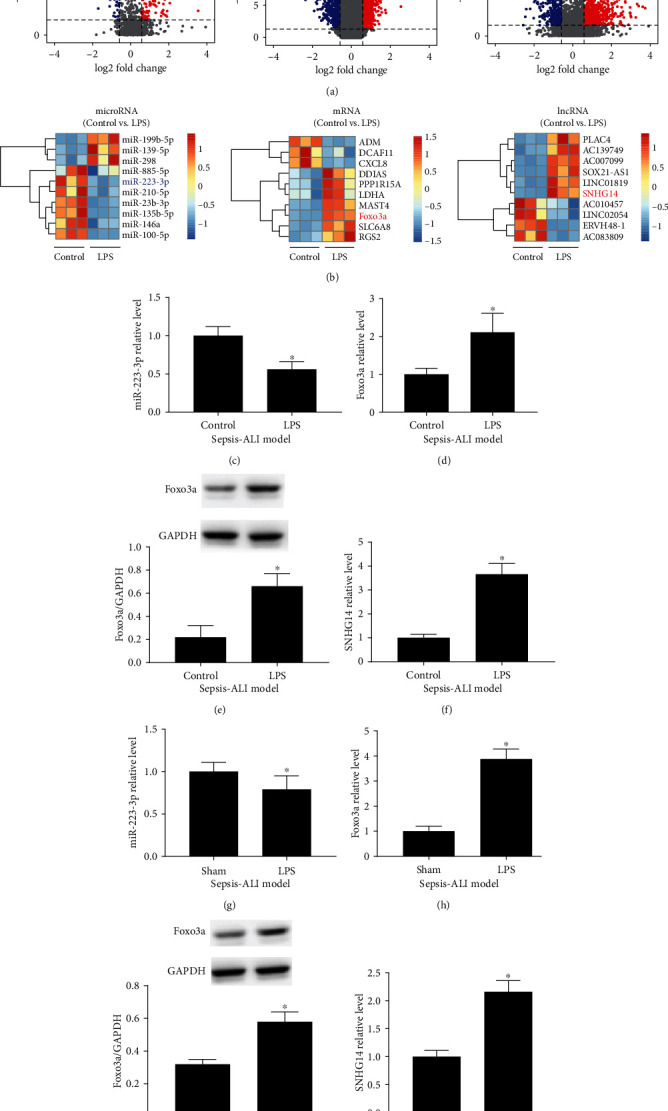
lncRNA-SNHG14/miR-223-3p/Foxo3a expression level altered in LPS-treated A549 cells and lung tissue collected from LPS-induced ALI models. (a) RNA-sequence analysis was used to reveal the microRNAs (left), mRNAs (middle), and lncRNAs (right) profiles in A549 cells treated by LPS for 24 h. Differentially expressed genes were shown in the volcano plot. According to the adjusted *P* value, the top ten differentials expressed microRNAs (left), mRNAs (middle), and lncRNAs (right) were shown in heat maps. A549 cells were treated by LPS for 24 h. miR-223-3p (c), Foxo3a mRNA (d), Foxo3a protein (e), and lncRNA-SNHG14 (f) were detected using qRT-PCR and western blot, respectively. Lung tissue samples were collected from LPS-induced ALI models. miR-223-3p (g), Foxo3a mRNA (h), Foxo3a protein (i), and lncRNA-SNHG14 (j) were measured. ^∗^*P* < 0.05 compared with the control or Sham group.

**Figure 2:**
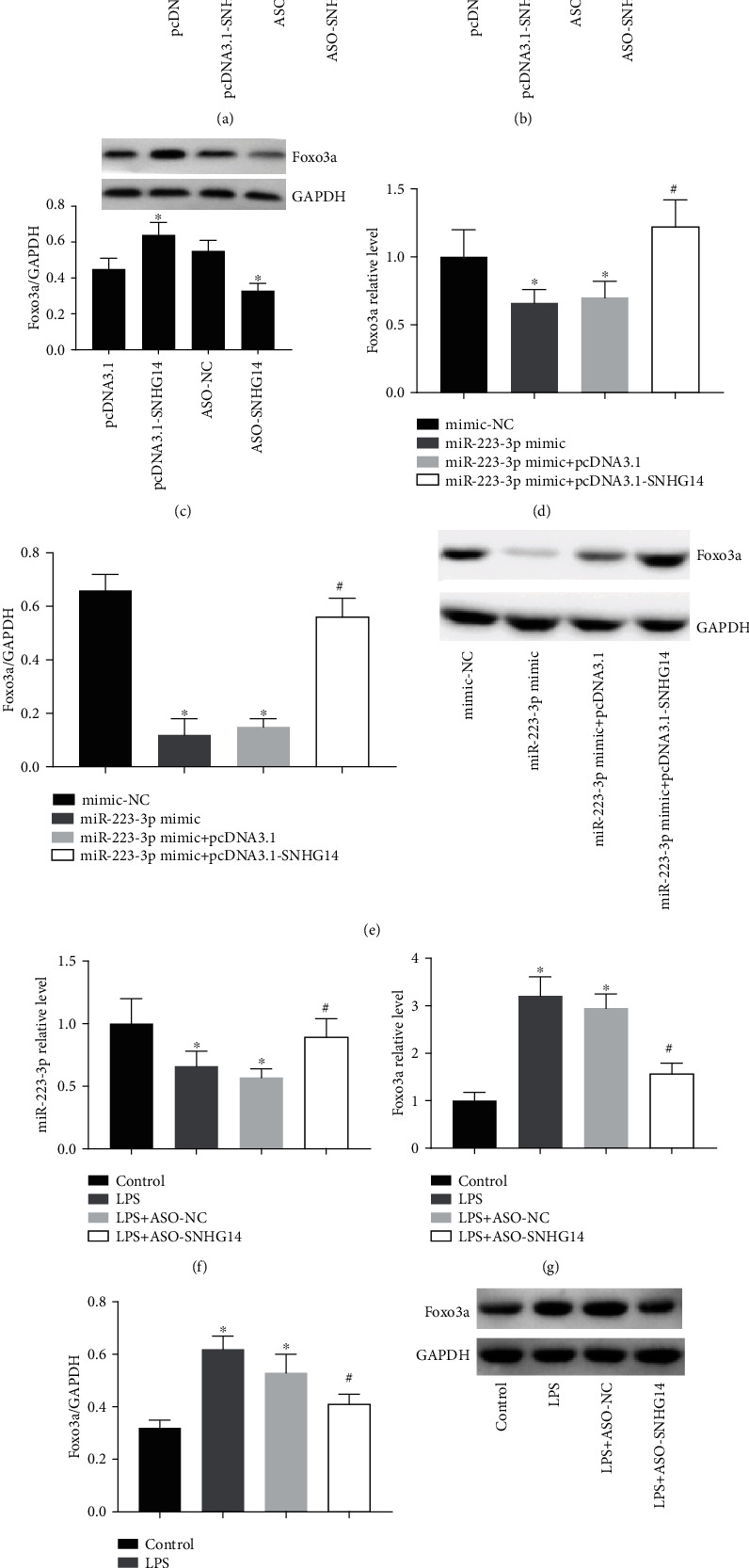
lncRNA-SNHG14 regulated expression of miR-223-3p/Foxo3a in A549 cells. pcDNA3.1-SNHG14 and ASO-SNHG14 were transfected into A549 cells. After 48 h, miR-223-3p (a), Foxo3a mRNA (b), and Foxo3a protein (c) by qRT-PCR and western blot. miR-223-3p was transfected or cotransfected with pcDNA3.1-SNHG14, Foxo3a mRNA (d), and Foxo3a protein (e) were detected by qRT-PCR and western blot. ASO-SNHG14 was transfected into cells 24 h ahead of LPS treatment. Under LPS treating condition, miR-223-3p (f), Foxo3a mRNA (g), and Foxo3a protein (h) were detected. ^∗^*P* < 0.05 compared with transfection negative control or LPS untreated control. #*P* < 0.05 compared with miR-223-3p mimic+pcDNA3.1 or LPS + ASO-NC.

First, we constructed an *in vitro* LPS-induced alveolar type II epithelial cell injury model using the A549 cell line and performed RNA sequencing to obtain a profile of LPS-induced transcriptomic changes. The sequencing results showed that there were 1162 lncRNAs with differential expression, 120 microRNAs with differential expression, and 1162 mRNAs with differential expression in A549 cells 24 hours after LPS treatment (Figure 1(a)). According to adjusted *P* value, the top 10 differentially expressed lncRNAs, microRNAs, and mRNAs were selected and shown in heat maps (Figure 1(b)). Then, we performed gene ontology (GO) and pathway enrichment analysis for the target genes of the top 10 (by *P* value) changed microRNA list that obtained from Figure 1(b) (left panel). As shown in Figure S2A, the chemokine signaling pathway can be found to be involved, which is consistent with the effect of LPS treatment. FoxO signaling pathway and mTOR signaling pathway, which are related to autophagy regulation, were also involved. A similar analysis was performed for all differential expressed mRNAs (DEGs) as shown in Figure 1(a). Chemokine-related GO biological processes and KEGG pathway terms were enriched (Figure S2B-C). Cluster analysis results for enriched terms are shown in FigureS2D. The protein-protein networks of DEGs are shown in Figure S1 E.

Based on the principle of ceRNA, we performed bioinformatics predictions on the top 10 differentially expressed lncRNAs, microRNAs, and mRNAs to evaluate the complementary binding potential of their sequences. We found that there may be a ceRNA interaction relationship between lncRNA-SNHG14, miR-223-3p, and Foxo3a. In RNA-seq results, lncRNA-SNHG14 and Foxo3a were upregulated in A549 cells treated with LPS, while miR-223-3p showed downregulation. Subsequently, we used qRT-PCR and western blot analysis to verify the expression of lncRNA-SNHG14, miR-223-3p, and Foxo3a in alveolar type II epithelial cells after LPS treatment *in vitro*. These results were consistent with the sequencing data (Figures 1(c)–1(f)). We also collected lung tissues of mouse ALI models. Compared to control tissues, the lung tissues of mouse ALI models displayed differences in the expression of lncRNA-SNHG14, miR-223-3p, and Foxo3a, with the differential expression patterns matching those observed *in vitro* (Figures 1(g)–1(k)).

lncRNA-SNHG14 in alveolar type II epithelial cells regulated the expression of miR-223-3p/Foxo3a as a ceRNA.

### 3.2. lncRNA-SNHG14 Regulate the Expression of miR-223-3p/Foxo3a as a ceRNA in Alveolar Type II Epithelial Cells

Subsequently, we tested if the ceRNA regulatory network composed of lncRNA-SNHG14/miR-223-3p/Foxo3a was present in alveolar type II epithelial cells. After overexpressing lncRNA-SNHG14 in A549 cells, the expression of miR-223-3p was decreased, while the mRNA and protein levels of Foxo3a were upregulated. After downregulating lncRNA-SNHG14, the expression of miR-223-3p and Foxo3a had an opposite change as aforementioned (Figures 2(a)–2(c)). Transfection of miR-223-3p mimic reduced the expression of Foxo3a mRNA and protein (Figures 2(d) and 2(e)), but lncRNA-SNHG14 overexpression partly restored the expression of Foxo3a. Moreover, downregulation of lncRNA-SNHG14 relieved LPS-induced level changes of miR-223-3p and Foxo3a (Figures 2(f) and 2(g)).

**Figure 3:**
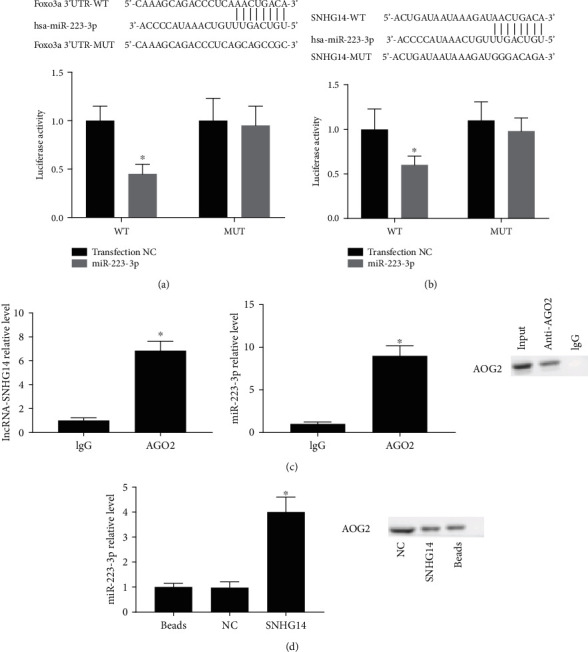
lncRNA-SNHG14 interacted with miR-223-3p/Foxo3a as a ceRNA. (a, b) The binding relationship of lncRNA-SNHG14/miR-223-3p and miR-223-3/Foxo3a was determined by dual-luciferase reporter assay. (c) In RIP assay, lncRNA-SNHG14 (left) and miR-223-3p (middle) levels in anti-AGO2 coprecipitates were measured by qRT-PCR.AGO2 binds that were detected by western blot (right). (d) In RNA pull-down assay, miR-223-3p level in biotin-labeled SNHG14 pull-down compounds was analyzed using qRT-PCR (left). AGO2 binds were detected by western blot (right). ^∗^*P* < 0.05 compared with transfection negative control or lgG or RNA pull-down control.

In a dual-luciferase reporter assay, a miR-223-3p mimic markedly reduced the luciferase activity of wt-SNHG14 and wt-3′UTR-Foxo3a (*P* < 0.05) in A549 cells but had no effects on that of mut-SNHG14 and mut-3′UTR-Foxo3a (Figures 3(a) and 3(b)). In an RNA immunoprecipitation (RIP) analysis, the mRNAs of lncRNA-SNHG14, miR-223-3p, and Foxo3a were enriched in the anti-Ago2 group, which suggested that lncRNA-SNHG14 could be used as ceRNA to regulate the expression of miR-223-3p/Foxo3a in A549 cells (Figure 3(c)). In the RNA pull-down analysis, AGO2 protein was present in the pull-down complex of SNHG14, and the expression of miR-223-3p was significantly enriched in the pull-down compound of SNHG14 (Figure 3(d)).

### 3.3. lncRNA-SNHG14 Participated in LPS-Induced Alveolar Type II Epithelial Cell Injury *In Vitro* and in a Murine LPS-Induced ALI Model by Regulating miR-223-3p/Foxo3a

**Figure 4:**
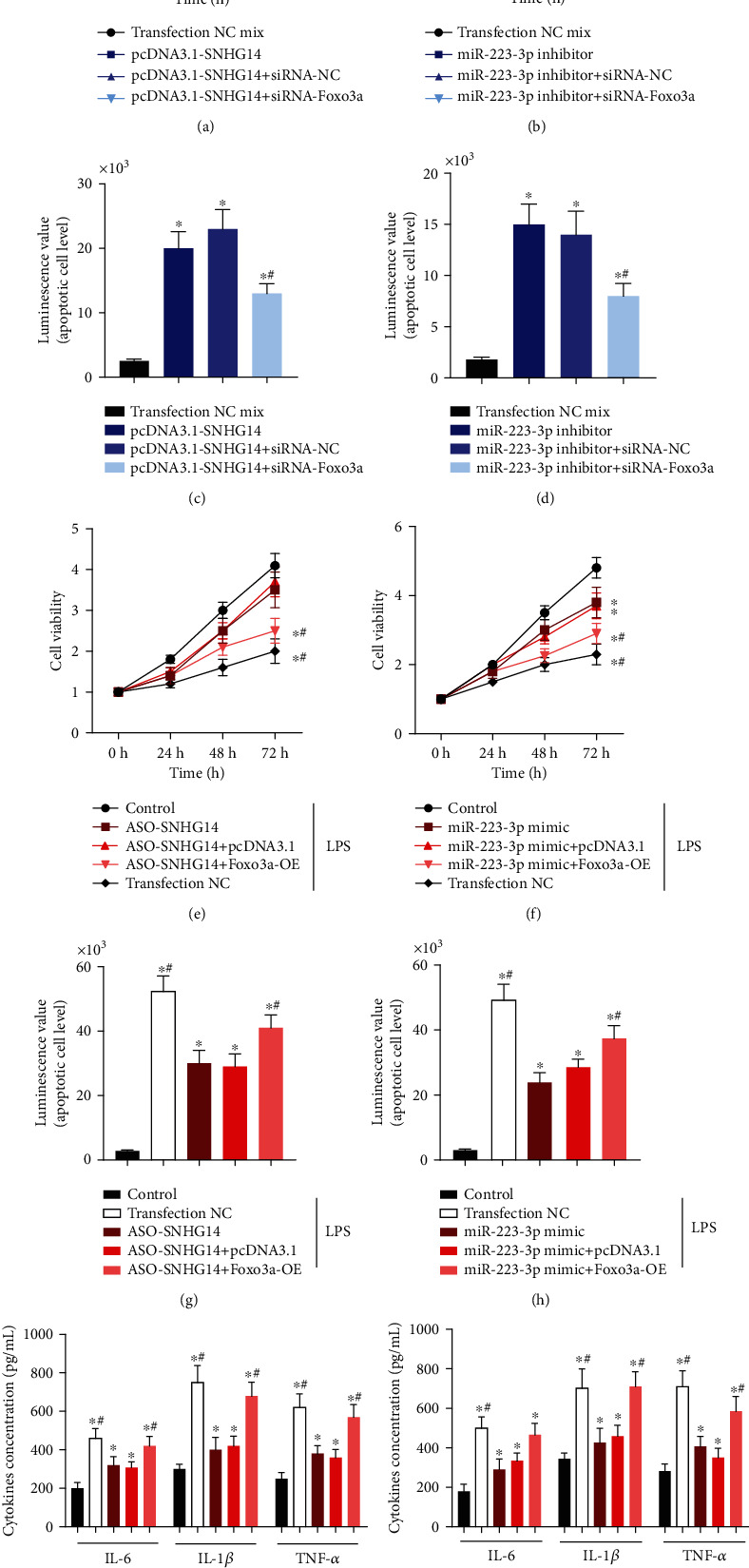
lncRNA-SNHG14/miR-223-3p/Foxo3a axis participated in LPS-induced A549 cells injury. A549 cells were transfected with pcDNA3.1-SNHG14, pcDNA3.1-SNHG14 + siRNA-Foxo3a, miR-223-3p, and miR-223-3p + siRNA-Foxo3a, respectively. Cell viability was detected using CCK-8 kit at 0, 24, 48, and 72 h (a, b). The apoptosis level of cells in each group was measured using RealTime-Glo™ Annexin V dye. Luminescence was detected using a fluorescence plate reader to indicate apoptosis (c, d). Cells were transfected with ASO-SNHG14, ASO-SNHG14 + pcDNA3.1-Foxo3a, miR-223-3p mimic, and miR-223-3p mimic+pcDNA3.1-Foxo3a, respectively. Cell viability (e, f), apoptotic cells (g, h), and cytokine level changes (i, j) were detected under LPS treating condition. ^∗^*P* < 0.05 compared with transfection negative control mix or LPS untreated control. ^#^*P* < 0.05 compared with cells without cotransfection of siRNA-Foxo3 or pcDNA3.1-Foxo3a.

We also analyzed the possible functions of lncRNA-SNHG14, miR-223-3p, and Foxo3a in the LPS-induced alveolar type II epithelial cell injury and ALI mouse model. In the A549 cells that were not treated with LPS, overexpression of lncRNA-SNHG14 or knockdown of miR-223-3p caused inhibition of cell proliferation (Figures 4(a) and 4(b)) and induced apoptosis (Figures 4(c) and 4(d)). These effects were offset by siRNA-Foxo3a transfection. When ASO-lncRNA-SNHG14 or miR-223-3p mimics were transfected in A549 cells before LPS treatment, the inhibition of cell proliferation (Figures 4(e) and 4(f)) and apoptosis (Figures 4(g) and 4(h)) induced by LPS was reduced. And the levels of interleukin- (IL-) 6, IL-1*β*, and tumor necrosis factor alpha (TNF-*α*) in the supernatant were reduced, suggesting that downregulation of lncRNA-SNHG14 or upregulation of miR-223-3p had a protective effect on LPS-induced alveolar type II epithelial cell injury. This protective effect could be antagonized by overexpression of Foxo3a.

**Figure 5:**
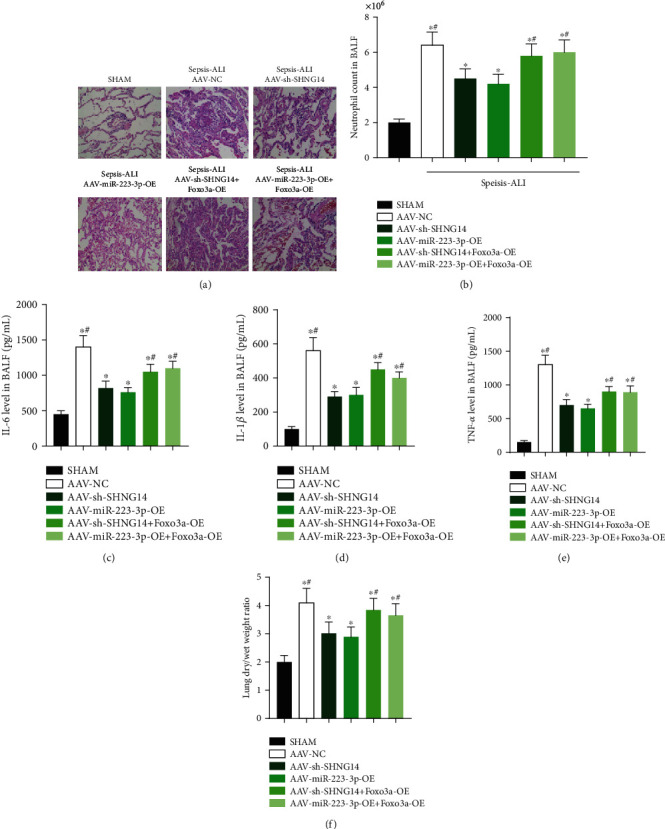
lncRNA-SNHG14/miR-223-3p/Foxo3a axis played a role in the LPS-induced ALI mice model. Animals were administered with AAV-SNHG14, AAV-miR-223-3p-OE, AAV-SNHG14 + AAV-Foxo3a-OE, and AAV-miR-223-3p-OE + AAV-Foxo3a-OE 2 weeks before LPS treatment, respectively. After 12 h of LPS instilling. Lung tissue samples were collected; lung injury level was estimated by pathological section ((a) hematoxylin-eosin staining). Bronchoalveolar lavage fluid (BALF) was collected. Neutrophil count (b), IL-6 (c), IL-1*β* (d), and TNF-*α* (e) levels in BALF were determined. (f) Lung wet/dry weight ratio was measured. ^∗^*P* < 0.05 compared with the Sham group; #*P* < 0.05 compared with the mice treated with AAV-SH-SNHG14 or AAV-miR-223-3p-OE alone.

In the murine LPS-induced ALI model, after knocking down lncRNA-SNHG14 or overexpressing miR-223-3p via AAVs, the lung injury of mice was relatively mild (Figure 5(a)). The neutrophil count, IL-6, IL-1*β*, and TNF-*α* levels in the bronchial lavage fluid were lower in ALI models with knocking down lncRNA-SNHG14 or overexpressing miR-223-3p (Figures 5(b)–5(f)). The lung W/D weight ratio was also lower than the ALI model that was only treated with AAV negative control (Figure 5(f)). These data suggested that knockdown of lncRNA-SNHG14 or overexpression of miR-223-3p had a protective effect on the lungs, and the protective effect of downregulation of lncRNA-SNHG14 or overexpression of miR-223-3p was offset by the overexpression of Foxo3a.

### 3.4. lncRNA-SNHG14/miR-223-3p/Foxo3a Mediated Autophagy in Alveolar Type II Epithelial Cells

**Figure 6:**
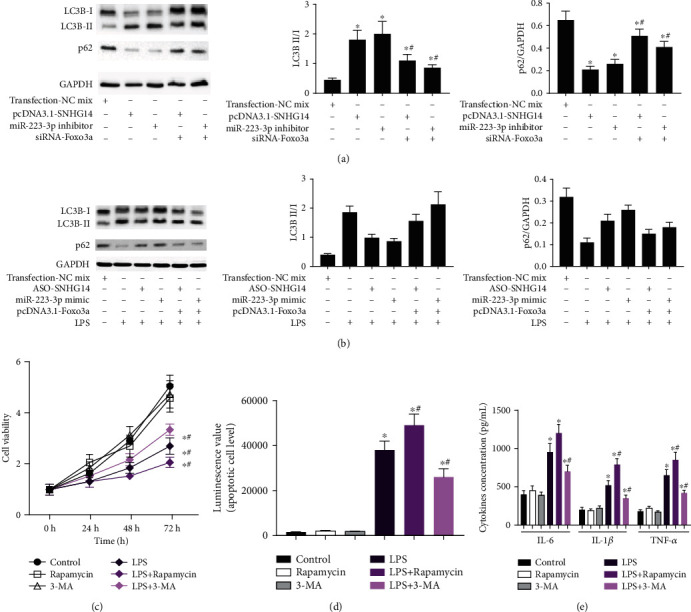
lncRNA-SNHG14/miR-223-3p/Foxo3a axis regulated autophagy in A549 cells. (a) A549 cells were transfected with pcDNA3.1-SNHG14, miR-223-3p inhibitor, pcDNA3.1-SNHG14 + siRNA-Foxo3a, and miR-223-3p inhibitor+siRNA-Foxo3a. Autophagy markers including LC3B II/I and p62 were detected using western blotting. (b) Cells were transfected with ASO-SNHG14, ASO-SNHG14 + pcDNA3.1-Foxo3a, miR-223-3p mimic, and miR-223-3p mimic+pcDNA3.1-Foxo3a, respectively. Autophagy markers were detected under LPS treating condition. (c) Cells were treated with rapamycin or 3-methyladenine. Cell viability (c), apoptosis (d), and cytokines (e) were measured under LPS treating condition. ^∗^*P* < 0.05 compared with transfection negative control or LPS untreated control; ^#^*P* < 0.05 compared with cells without cotransfection of siRNA-Foxo3 or pcDNA3.1-Foxo3a in (a, b). ^#^*P* < 0.05 compared with cell without treatment of rapamycin and 3-methyladenine in (c, e).

Reports have shown that miR-223-3p/Foxo3a is involved in the regulation of autophagy. Thus, the regulation of autophagy by lncRNA-SNHG14/miR-223-3p/Foxo3a was explored. In normal A549 cells, overexpression of lncRNA-SNHG14 or transfection of miR-223-3p inhibitors induced increasing LC3B II/I and reducing p62 level. It indicated activated autophagy. However, siRNA-Foxo3a transfection could impede this effect (Figure 6(a)). LPS treatment could induce autophagy. Knockdown of lncRNA-SNHG14 or upregulation of miR-223-3p could alleviate the activating of autophagy induced by LPS. This effect could also be partly antagonized by the upregulation of Foxo3a (Figure 6(b)). Finally, we analyzed the role of autophagy in LPS-induced alveolar type II epithelial cell injury. After using rapamycin (100 nM) to induce autophagy or 3-methyladenine to inhibit autophagy, the inhibition of cell proliferation and the levels of IL-6, IL-1*β*, and TNF-*α* in culture supernatant were analyzed. The rapamycin and 3-MA themselves had no effect on proliferation and apoptosis at the concentration indicated. The results suggested that induction of autophagy aggravated LPS injury in the cells, and inhibition of autophagy reduced the LPS injury in the cells (Figures 6(c)–6(e)). Thus, the lncRNA-SNHG14/miR-223-3p/Foxo3a axis is involved in LPS-induced injury by mediating the level of autophagy ongoing in alveolar type II epithelial cells.

## 4. Discussion

ALI is one of the most fatal complications in patients with sepsis. If it is not controlled, it progresses into ARDS [17, 18]. ALI is an important reason for the high mortality of patients with sepsis in intensive care units. Inflammatory injury of alveolar type II epithelial cells, lung capillary endothelial cells, and lung macrophages is the main pathophysiological change during the process of ALI [19]. Increasing studies have focused on the role of lncRNAs in ALI caused by sepsis. For example, Qiu and XuY showed that lncRNA-TUG1 was downregulated in an ALI mouse model. Overexpression of lncRNA-TUG1 reduced the damage in lung capillary endothelial cells [20]. The possible mechanism may be that TUG1 alleviated LPS-induced apoptosis and inflammation via targeting miR-34b-5p/GAB1. Chen et al. showed that lncRNA-THRIL was significantly upregulated in a sepsis-induced ALI mouse model. Knockdown of lncRNA-THRIL effectively reduced the lung injury in mice and inhibited the release of factor-*α*, IL-1*β*, IL-6, and other cytokines. In their *in vitro* experiments, lncRNA-THRIL upregulated ROCK2 levels through sponging miR-424 to promote the apoptosis of vascular endothelial cells and the release of inflammatory cytokines [21]. Wang et al. reported that downregulation of lncRNA CASC9 inhibited LPS-induced injury in human small airway epithelial cells by regulating the miR-195-5p/PDK4 axis [22]. lncRNA-5657 has been shown to be highly expressed in the airway lavage fluid cells of ARDS patients. High lncRNA-5657 expression was also detected in a CLP-induced sepsis rat model. The *in vitro* silencing of lncRNA-5657 alleviated sepsis-induced injury in rat alveolar macrophages by suppressing the expression of spinster homology protein 2 [23]. lncRNA-MALAT1 was shown to be used as a ceRNA to regulate miR-149/MyD88 to promote inflammatory responses of LPS-induced ALI [24]. Alveolar type II epithelial cells are an important basis for alveolar structure and function. Revealing the molecular mechanism of alveolar type II epithelial cell injury during ALI in patients with sepsis is of great help to the prevention and treatment of ALI. Zhou et al. reported that lncRNA NEAT1 regulated HMGB1-RAGE signaling in LPS-induced alveolar epithelial cell injury [15]. However, the role of lncRNA in alveolar epithelial cells still needs to be further studied in the process of sepsis-induced ALI.

This study used the non-small-cell lung cancer cell line, A549, derived from alveolar type II epithelial cells and treated the cells with LPS to simulate the alveolar cell injury caused by sepsis. RNA-sequencing analysis revealed a series of lncRNA, microRNA, and mRNA changes in the A549 cells after LPS treatment. Among them, the upregulation of lncRNA-SNHG14 aroused our attention. We also confirmed the upregulation of lncRNA-SNHG14 in LPS-treated primary mouse alveolar type II epithelial cells and LPS-induced ALI mouse models. Some previous studies have confirmed that inhibition of the lncRNA-SNHG14 expression protects some cells under stress from injury. For example, Lu et al. found that knockdown of lncRNA-SNHG14 reduced the cardiomyocyte damage caused by hypoxia. The mechanism was related to the regulation of apoptosis by lncRNA-SNHG14 through the miR-25-3p/KLF4 axis [25]. In the ischemia-reperfusion injury mouse model, overexpression of lncRNA-SNHG14 offsets the protective effect of dexmedetomidine on the spinal cord, suggesting that lower lncRNA-SNHG14 levels were beneficial to resisting spinal cord ischemia-reperfusion injury [26]. In this study, we found that overexpression of lncRNA-SNHG14 inhibited A549 cell proliferation, and knockdown of lncRNA-SNHG14 reduced the cell injury caused by LPS. Combined with the evidence in this study, we believe that overexpression of lncRNA-SNHG14 induced by LPS may promote alveolar type II epithelial cell injury.

We then looked for the possible molecular mechanism of lncRNA-SNHG14. The ceRNA theory integrates the changes in lncRNAs, microRNAs, and mRNAs to explain the phenotypic changes in the cells. Therefore, based on the RNA-sequencing data, we searched among microRNAs and mRNAs with differential expression for targets that may be associated with lncRNA-SNHG14. Finally, we showed that miR-223-3p and lncRNA-SNHG14 had a complementary sequence region, and they were downregulated in LPS-treated A549 cells. In addition, the target gene of miR-223-3p, Foxo3a, was upregulated in the LPS-treated A549 cells. In previous *in vitro* experiments, we confirmed that lncRNA-SNHG14 regulated the expression of miR-223-3p/Foxo3a. The dual-luciferase reporter assay and RIP analysis also confirmed that this regulation was based on ceRNA theory. Liu et al. reported that miR-223 reduces sepsis-induced lymphocyte apoptosis [27]. Tan et al. found that in LPS-induced renal tubular epithelial cell injury, the expression of miR-223-3p is insufficient, leading to an increase in NLRP3, which can induce pyroptosis in cells [28]. In this study, we found that miR-223-3p had the opposite function of lncRNA-SNHG14 in LPS-induced injury in A549 cells and in the ALI mouse model, i.e., miR-223-3p reduces the damage caused by LPS. This was consistent with previous studies where miR-223-3p offered protection against sepsis-induced injury. More importantly, changing the expression of Foxo3a partially offsets the function of lncRNA-SNHG14 or miR-223-3p, suggesting that the lncRNA-SNHG14/miR-223-3p/Foxo3a axis not only existed in alveolar type II epithelial cells but also participated in the cell injury caused by LPS.

Therefore, this study also investigated the regulation of autophagy by the lncRNA-SNHG14/miR-223-3p/Foxo3a axis. As expected, lncRNA-SNHG14 promoted autophagy in the A549 cells through the miR-223-3p/Foxo3a axis. The target gene of miR-223-3p, Foxo3a, is an important autophagy regulatory gene that induces autophagy. Zhou et al. reported that miR-223-3p inhibited doxorubicin-induced autophagy by targeting Foxo3a to silence [29]. Foxo3a is a multifunctional transcription factor, which can respond to environmental stress and lead to changes in target genes, resulting in alteration in biological activities. Foxo3a coordinately increases autophagy by binding directly to the promoters of autophagy-related genes, including ATG12, Beclin1, and LC3 under stress condition [30]. After LPS treatment, upregulation of Foxo3a would induce extra autophagy which resulting in loss of alveolar epithelial cells [10]. This may explain why overexpression of Foxo3a can counteract the protection of SNHG14. Of course, studies have also shown that lncRNA-SNHG14 promoted autophagy through other pathways. For example, lncRNA-SNHG14 was shown to stimulate cell autophagy to facilitate cisplatin resistance of colorectal cancer by regulating the miR-186/ATG14 axis [31]. In this study, we showed that the promotion of autophagy led to the aggravation of LPS-induced A549 cell injury while inhibiting autophagy showed a protective effect. These results may only apply to our models because the role of autophagy in lung injury caused by LPS is currently controversial. Some studies have suggested that insufficient autophagy reduces the tolerance of cells to the inflammatory response, while other studies have suggested that excessive autophagy promotes cell death. Further research is needed to reveal whether the lncRNA-SNHG14/miR-223-3p/Foxo3a axis plays a role in LPS-induced injury in other lung cells, such as alveolar macrophages and pulmonary microvascular endothelial cells, whether this axis regulates autophagy and the role of autophagy in LPS-induced cell injury. Since pulmonary endothelial cells (PECs) are essential part of the alveolar structure, when lung injury occurs, PECs are often the first to be affected. Severe PEC injury will lead to the pulmonary blood barrier permeability barrier, causing diffuse lung interstitial and alveolar edema and affecting respiratory function. Therefore, PEC protection has the significance of preventing and treating ALI. But our study ignored endothelial cells due to limited attention. This is the limitation of this study, and we will focus on endothelial cells in further studies.

## 5. Conclusions

Collectively, this study showed that the change in expression of the lncRNA-SNHG14/miR-223-3p/Foxo3a axis existed in both an LPS-induced alveolar type II epithelial cell injury model and a murine ALI model. lncRNA-SNHG14 could be used as a ceRNA to regulate miR-223-3p/Foxo3a, thereby controlling the occurrence of autophagy and ultimately participating in LPS-induced alveolar type II epithelial cell injury. Intervention with the lncRNA-SNHG14/miR-223-3p/Foxo3a axis may provide a therapeutic strategy for ALI/ARDS.

## Data Availability

The data used to support the findings of this study are available from the corresponding author upon request.

## Supplementary Materials

Supplementary Materials Figure S1: effect of the transfection/infective agent used in the present study. After transfection with pcDNA3.1-SNHG14 (a), ASO-SNHG14 (b), miR-223-3p mimic (c), miR-223-3p inhibitor (d), pcDNA3.1-FoxO3a (e), siRNA-FoxO3a (f), AAC-sh-snHG14 (g), AAV-miR-223-3p-OE (h), and AAV-FoxO3a-OE (i), the expression of target genes were verified by qRT-PCR. ^∗^*P* < 0.05 compared with negative transfection control. Figure S2: gene functional and pathway enrichment analysis of differentially expressed microRNAs and mRNAs in LPS-treated A549 cells. (a) Top ten results of signal pathways enrichment analysis among target genes of top ten differentials expressed microRNAs. (b, c) annotation analysis of GO Biological Process and enrichment analysis of KEGG pathway of differential expressed mRNAs.
