# Supplementary material for: lncRNA-SNHG14 Plays a Role in Acute Lung Injury Induced by Lipopolysaccharide through Regulating Autophagy via miR-223-3p/Foxo3a
Source: Mediators Inflamm. 2021 Sep 8;2021:7890288. doi: 10.1155/2021/7890288 (PMC8443345; doi:10.1155/2021/7890288)
Supplement: Supplementary Materials — Figure S1: effect of the transfection/infective agent used in the present study. After transfection with pcDNA3.1-SNHG14 (a), ASO-SNHG14 (b), miR-223-3p mimic (c), miR-223-3p inhibitor (d), pcDNA3.1-FoxO3a (e), siRNA-FoxO3a (f), AAC-sh-snHG14 (g), AAV-miR-223-3p-OE (h), and AAV-FoxO3a-OE (i), the expression of target genes were verified by qRT-PCR. ∗P < 0.05 compared with negative transfection control. Figure S2: gene functional and pathway enrichment analysis of differentially expressed microRNAs and mRNAs in LPS-treated A549 cells. (a) Top ten results of signal pathways enrichment analysis among target genes of top ten differentials expressed microRNAs. (b, c) annotation analysis of GO Biological Process and enrichment analysis of KEGG pathway of differential expressed mRNAs. [file 7890288.f1.docx]

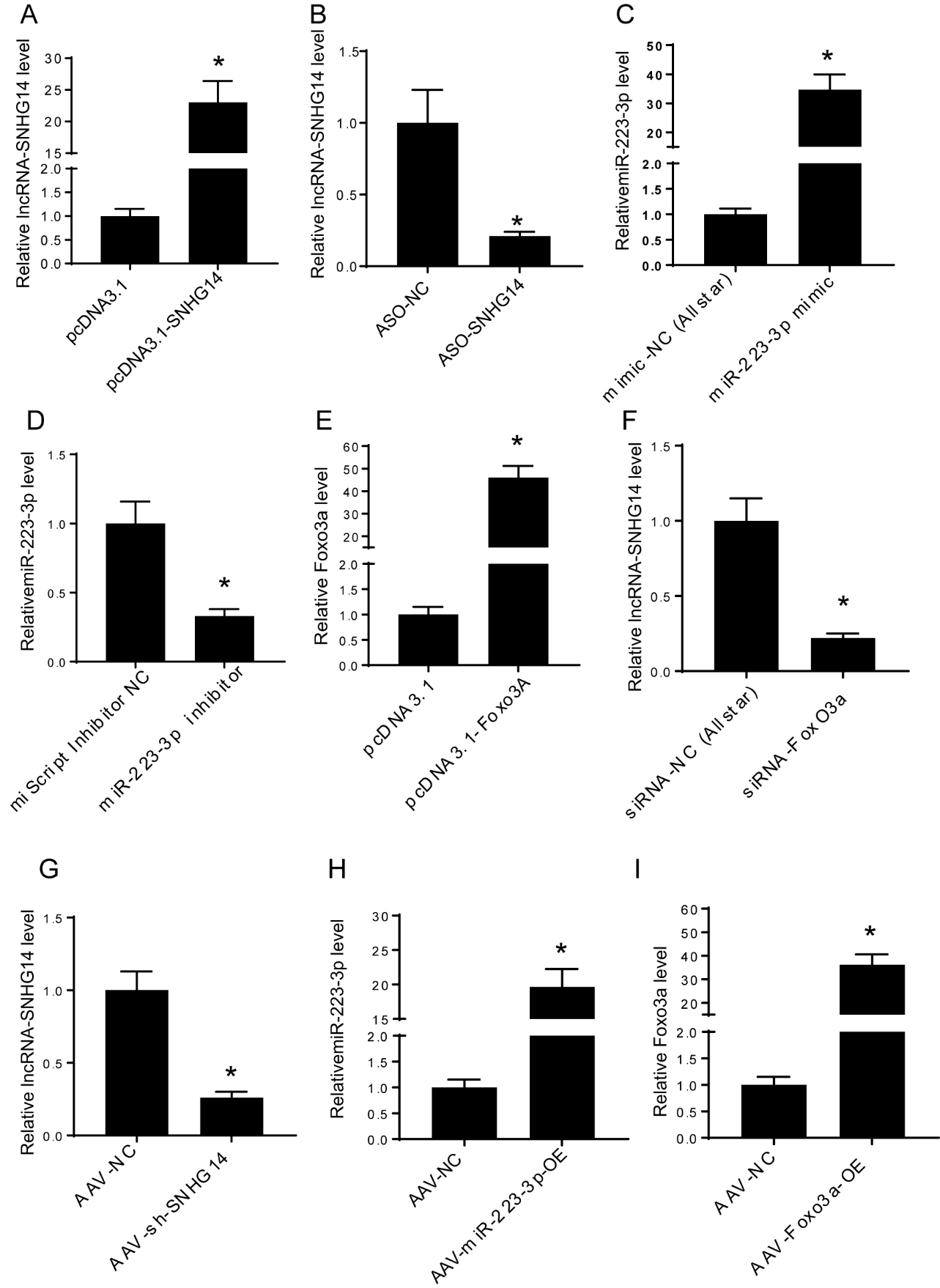


Figure S1 Effect of the transfection/infective agent used in the present study. After transfection with pcDNA3.1-SNHG14 (A), ASO-SNHG14 (B), miR-223-3p mimic (C), miR-223-3p inhibitor (D), pcDNA3.1-FoxO3a (E), siRNA-FoxO3a (F), AAC-sh-snHG14 (G), AAV-miR-223-3p-OE (H) and AAV-FoxO3a-OE (I), the expression of target genes were verified by qRT-PCR.**P*<0.05 compared with negative transfection control.


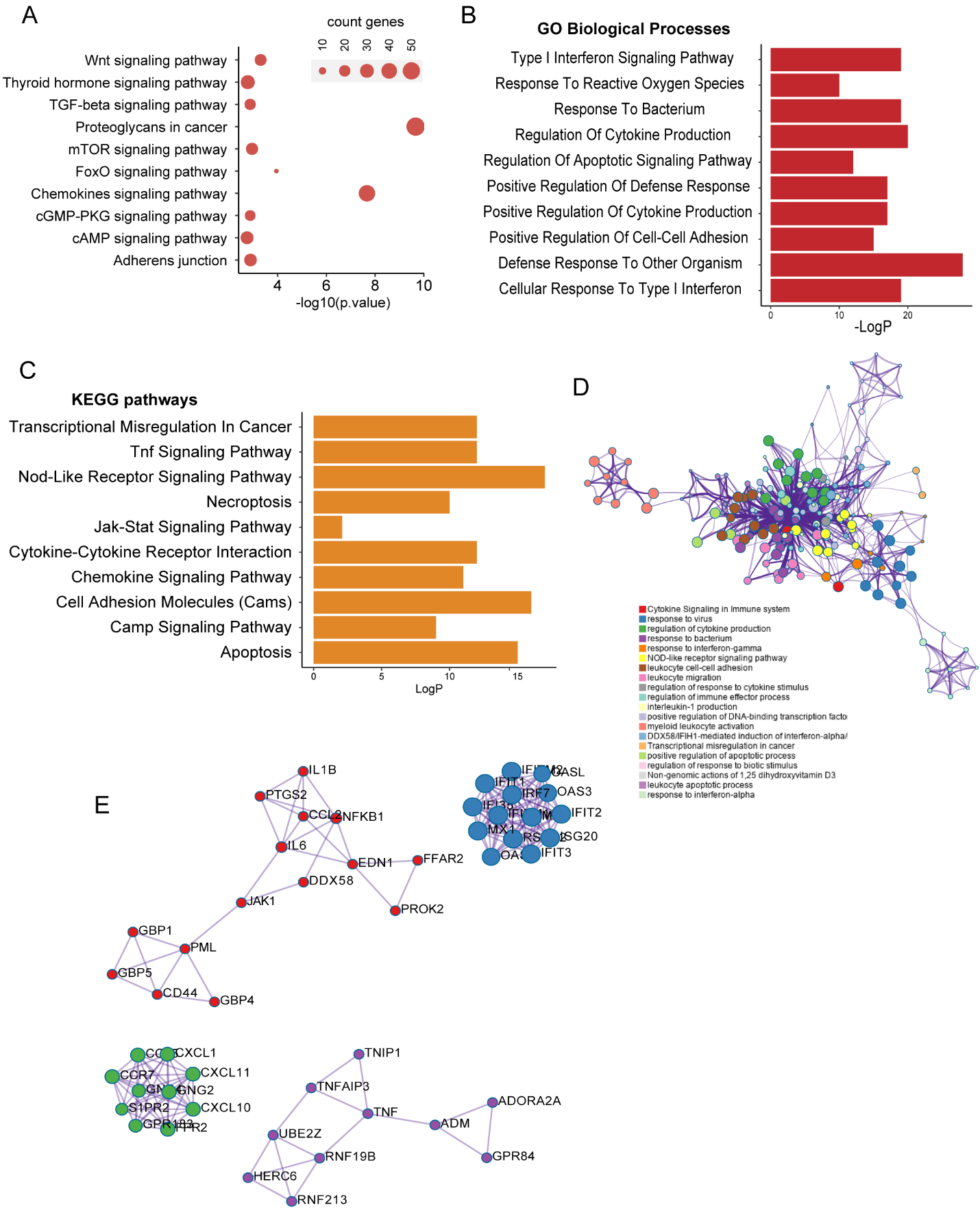


Figure S2 Gene functional and pathway enrichment analysis of differentially expressed microRNAs and mRNAs in LPS-treated A549 cells. (A) Top ten results of signal pathways enrichment analysis among target genes of top ten differentials expressed microRNAs. (B-C) annotation analysis of GO Biological Process and enrichment analysis of KEGG pathway of differential expressed mRNAs. (D) Cluster analysis for enriched GO Biological Process and KEGG pathway terms. (F) Protein-protein interaction networks for proteins coded by differential expressed mRNAs.
